# The molecular mechanism of contrast-induced nephropathy (CIN) and its link to in vitro studies on iodinated contrast media (CM)

**DOI:** 10.1051/bmdcn/2018080101

**Published:** 2018-02-26

**Authors:** Jai-Sing Yang, Yan-Ru Peng, Shih-Chang Tsai, Yeu-Sheng Tyan, Chi-Cheng Lu, Hong-Yi Chiu, Yu-Jen Chiu, Sheng-Chu Kuo, Yuh-Feng Tsai, Ping-Chin Lin, Fuu-Jen Tsai

**Affiliations:** 1 Department of Medical Research, China Medical University Hospital, China Medical University Taichung 404 Taiwan; 2 School of Medicine, China Medical University Taichung 404 Taiwan; 3 Department of Biological Science and Technology, China Medical University Taichung 404 Taiwan; 4 Department of Medical Imaging, Chung Shan Medical University Hospital Taichung 402 Taiwan; 5 School of Medical Imaging and Radiological Sciences, Chung Shan Medical University Taichung 402 Taiwan; 6 School of Medicine, Chung Shan Medical University Taichung 402 Taiwan; 7 Department of Pharmacy, Buddhist Tzu Chi General Hospital Hualien 970 Taiwan; 8 Division of Reconstructive and Plastic Surgery, Department of Surgery, Taipei Veterans General Hospital Taipei 112 Taiwan; 9 Chinese Medicinal Research and Development Center, China Medical University Hospital, China Medical University Taichung 404 Taiwan; 10 School of Pharmacy, China Medical University Taichung 404 Taiwan; 11 Department of Diagnostic Radiology, Shin-Kong Wu Ho-Su Memorial Hospital Taipei 111 Taiwan; 12 School of Medicine, Fu-Jen Catholic University Taipei 242 Taiwan; 13 Department of Medical Imaging, Chia-Yi Christian Hospital Chiayi 600 Taiwan; 14 Genetics Center, Department of Medical Research, China Medical University Hospital Taichung 404 Taiwan; 15 School of Chinese Medicine, China Medical University Taichung 404 Taiwan; 16 Department of Medical Genetics, China Medical University Hospital Taichung 404 Taiwan

**Keywords:** Iodinated contrast media, The management of anaphylactic reaction by iodinated contrast media, Dose for non-ionic contrast media, Contrast-induced nephropathy (CIN), *In vitro* studies on iodinated contrast media

## Abstract

Iodinated contrast media (iodinated CM) have increased ability to absorb x-rays and to visualize structures that normally are impossible to observe in a radiological examination. The use of iodinated CM may destory renal function, commonly known as contrast-induced nephropathy (CIN), which can result in acute renal failure (ARF). This review article mainly focuses on the following areas: (1) classifications of iodinated CM: ionic or non-ionic, high-osmolarity contrast media (HOCM), low-osmolarity contrast media (LOCM) and iso-osmolarity contrast media (IOCM); (2) an introduction to the physical and chemical properties of the non-ionic iodinated CM; (3) the management of anaphylactic reaction by iodinated CM; (4) a suggested single injection of adult doses and maximum dose for non-ionic iodinated CM; (5) the molecular mechanism of contrast-induced nephropathy (CIN); (6) *In vitro* studies on iodinated CM. Based on above information, this review article provide an insight for understanding the drug safety of iodinated CM.

## 1. Introduction

Iodinated contrast media (iodinated CM) absorb x-rays and visualize structures that are normally hard to observe in a radiological examination [1-4]. It has been used widely for many years. Contrast media provide an ability to enhance normal structures or pathological lesions, which makes these places look different from surrounding. The mechanism of iodinated contrast media is based on shielding effect: high energy x-ray penetrates substances and yields a dark place in a plane image. Iodine, the content of iodinated contrast media, absorbs the energy of x-ray; that is to say, iodinated CM “shield” x-ray from detector and lead to a high density, white “shadow” appearing. Iodinated CM elevate the sensitivity and diagnostic accuracy in radiological examination [1, 5-7].

**Fig. 1:**
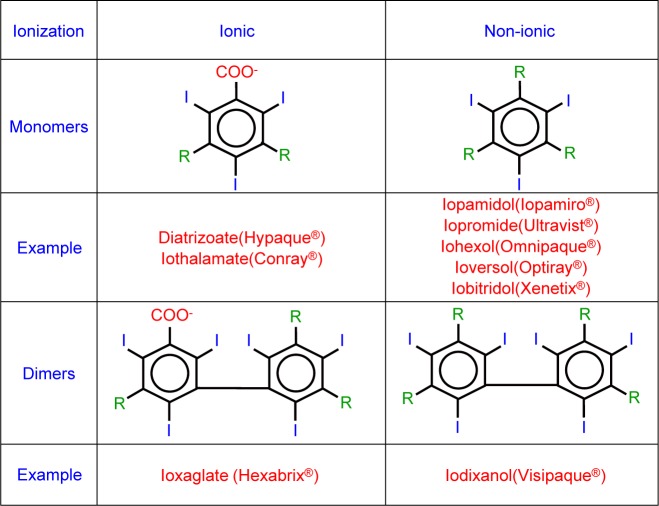
Water-soluble iodinated CM are divided into four groups based on the structure. They are ionic monomer, ionic dimer, nonionic monomer and nonionic dimer.

**Fig. 2:**
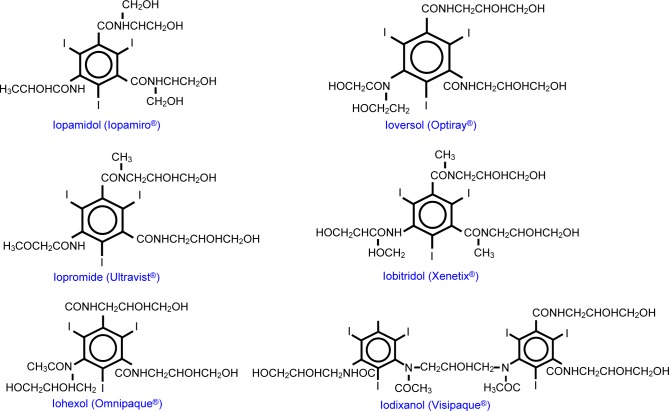
The chemical structures of currently used non-ionic iodinated CM.

Based on the solubility, iodinated CM are divided into three groups: oily iodinated CM, water-soluble iodinated CM and water-insoluble iodinated CM [8-10]. Iodinated CM are usually classified into ionic iodinated CM and non-ionic iodinated CM [10, 11]. Generally, ionic contrast media have higher osmolality, higher toxicity and higher anaphylactic reaction. Non-ionic contrast media possess lower osmolality, lower toxicity and lower anaphylactic reaction [12, 13]. Based on the structure, iodinated CM are divided into four groups: ionic monomer, ionic dimer, nonionic monomer and nonionic dimer (Fig. 1). Based on the osmolality, iodinated CM are classified into high-osmolar contrast media (HOCM), low-osmolar contrast media (LOCM) and isoosmolar contrast media (IOCM). High-osmolar contrast media (HOCM) is characterized by osmolarity of above 1500 mOsm/kg H_2_O. Low osmolar contrast media (LOCM) is characterized by osmolarities within a relatively wide range of 300-900 mOsm/kg H_2_O. The iso-osmolar contrast media (IOCM) is characterized by osmolarity level similar to that of blood (290 mOsm/kg H_2_O) [14, 15]. The osmolarity of high-osmolar contrast media (HOCM) is up to 7 or 8 fold greater than blood and has been associated with high risk of adverse drug reactions (ADR) and renal toxicity. Since the late 1960s, the nonionic low-osmolar contrast media (LOCM) have been developed to better safety and replace ionic iodinated CM for clinical uses. In 1996, the US Food and Drug Administration (FDA) approved the iso-osmolar contrast media (IOCM), iodixanol (Visipaque^®^), to have a better safety profile [14]. Furthermore, discomfort such as pain and heat associated with the injection site was found to be lower when using iso-osmolarity contrast media (IOCM) than low osmolar contrast media (LOCM) [14]. It is low neuro-toxicity and low osmolality that are important to intrathecal route injected contrast media, such as Iopamidol (Iopamiro^®^) 300 and Iohexol (Omnipaque^®^) 300 [16, 17]. Table 1 shows the biologic adverse drug reaction (ADR) difference between ionic iodinated CM and non-ionic iodinated CM. Currently used non-ionic iodinated CM in Taiwan and their chemical properties are summarized in Table 2. The chemical structures of non-ionic iodinated CM are shown in Fig. 2 [3, 11, 18-31]. In Table 3, we summarized the suggested single injection of adult doses and maximum dose for non-ionic iodinated CM by intra-arterial route. In Table 4, we summarized the suggested single injection of adult doses and maximum dose for non-ionic iodinated CM by intravenous route. In Table 5, we summarized the suggested single injection of adult doses and maximum dose for non-ionic iodinated CM by intrathecal route.

**Table 1:** The biologic adverse reaction between ionic and non-ionic contrast media.

Biologic adverse reaction	Ionic contrast media	Non-ionic contrast media
Thermal effect	Moderate	Mild to less
Pain during injection	Moderate	Mild to less
Nausea and vomiting	Moderate	Mild to less
Toxicity to kidney	Higher	Lower
Tissue necrosis when extravasation occurs	More severe	Less severe
Other allergic effects	Often (around 10%)	Seldom (lower than 5%)

**Table 2:** The chemistry and physical properties of non-ionic contrast media in Taiwan [31].

Brand name	Iopamiro	Ultravist	Omnipaque	Optiray	Xenetix	Visipaque
Generic name	Iopamidol	Iopromide	Iohexol	Ioversol	Iobitridol	Iodixanol
Iodine concentration (mgI/*ml*)	200 250 300 (Taiwan) 370 (Taiwan)	150 240 300 (Taiwan) 370 (Taiwan)	140 180 210 240 300 (Taiwan) 350 (Taiwan)	240 300 320 (Taiwan) 350 (Taiwan)	250 300 (Taiwan) 350 (Taiwan)	270 320 (Taiwan)
Osmolality (mOsmo/kg H_2_O, 37°C)	413 524 616 (Taiwan) 796 (Taiwan)	328 483 607 (Taiwan) 774 (Taiwan)	322 408 460 520 672 (Taiwan) 844 (Taiwan)	502 651 702 (Taiwan) 792 (Taiwan)	585 695 (Taiwan) 915 (Taiwan)	290 (Taiwan)
	Low osmolality	Low osmolality	Low osmolality	Low osmolality	Low osmolality	Iso-osmolality
Viscosity (mPa-s, 37°C)	2.0 3.0 4.7 (Taiwan) 9.4 (Taiwan)	1.5 2.8 4.9 (Taiwan) 10.0 (Taiwan)	1.5 2.0 2.5 3.4 6.3 (Taiwan) 10.4 (Taiwan)	3.0 5.5 5.8 (Taiwan) 9.0 (Taiwan)	4.0 6.0 (Taiwan) 10.0 (Taiwan)	11.8 (Taiwan)
Median lethal dose (LD_50_)	21.8 g I/Kg	18.5 g I/Kg	18.5 g I/Kg	17.0 g I/Kg	15.9 g I/Kg	17.9 g I/Kg
Expiration duration	5 years	3 years	3 years	3 years	3 years	3 years
National Health Insurance in Taiwan (NHI), 2017	Cover	Cover	Cover	Cover	Cover	Self-paid
Administration	Intravenous injection; intra-arterial injection; Intrathecal injection (Iopamiro 300, Omnipaque 300); Oral					Intravenous injection
Uses	Computed tomography (CT); Angiocardiography; Arteriography of cerebral arteries; Pyelography; Peripheral angiography					Angiocardiography Computed tomography (CT)

**Table 3:** Suggested single injection of adult doses and maximum total dose for non-ionic contrast media by intra-arterial injection [31].

Non-ionic contrast media		Angiography of arteries of extremity	Femoral arteriography	Aortography	Arteriography	Arteriography of cerebral arteries	Cardiac ventriculography, Left (FDA Dosage)	Cardiac ventriculography, Left (Off label Dosage)	Coronary angiography (FDA Dosage)	Coronary angiography (Off label Dosage)	Inferior vena cavogram
Iopromide (Ultravist) (300 mgI/*ml*)	Adult doses suggestion	5-40 *ml* for subclavian or femoral artery	65 *ml*			3-12 *ml* for carotid arteries					
		25-50 *ml* for aortic bifurcation				4-12 *ml* for vertebral arteries					
						20 to 50 *ml* for aortic arch injection					

	Maximum dose	250 *ml*				150 *ml*					

Iopromide (Ultravist) (370 mgI/*ml*)	Adult doses suggestion			Blood flow and vascular and pathological nature of the vessels of interest			30-60 *ml*	44-60 *ml*	3-14 *ml* for right or left coronary artery	7 to 10 *ml* (4-5 injections) -left coronary artery	Blood flow and vascular and pathological nature of the vessels of interest
										7-10 *ml* (2 to 3 injections)- right coronary artery	

	Maximum dose			225 *ml*			225 *ml*		225 *ml*		225 *ml*

Ioversol (Optiray) (320 mgI/*ml*)	Adult doses suggestion					2-12 *ml*	40 *ml* (30-50 *ml*)		45 *ml* (10-80 *ml*)		

	Maximum dose					200 *ml*					

Iobitridol (Xenetix) (350 mgI/*ml*)	Adult doses suggestion			10-80 *ml*			30-60 *ml*				

	Maximum dose			250 *ml*							

					Carotid arteries: 10-14 *ml*	10-14 *ml*					
					Verterbral arteries: 10-12 *ml*						
					Right coronary artery: 3-8 *ml*						
Iodixanol (Visipaque) (320 mgI/*ml*)	Adult doses suggestion				Left coronary artery: 3-10 *ml*						
					Left ventricle: 20-45 *ml*						
					Renal arteries: 8-18 *ml*						
					Aortography: 30-70 *ml*						
					Major aorta branch: 10-70 *ml*						
					Peripheral arteries: 15-30 *ml*						
					Aortofermoral runoffs: 20-90 *ml*						

	Maximum dose				250 *ml* (80 gI)	175 *ml* (80 gI)

**Table 4:** Suggested single injection of adult doses and maximum total dose for non-ionic contrast media by Intravenous injection [31].

Non-ionic contrast media		Computerized axial tomography, Body	Computerized axial tomography of head (brain)	Computerized axial tomography of abdomen	Intravenous pyelogram (urography)	Angiocardiography-Coronary Arteriography/Ventriculography	Angiocardiography-ventriculography or nonselective opacification of multiple coronary arteries	Aortography	Arteriography, peripheral	Arteriography, selective visceral	Arteriography of cerebral arteries	Renal arteriography	Venography
Iopromide (Ultravist) (300 mgI/*ml*)	Adult doses suggestion	50-200 *ml* for bolus IV injection	50-200 *ml*		300 mgI/kg								
		100-200 *ml* for rapid IV infusion											

	Maximum dose	200 *ml* (60 gI)	200 *ml* (60 gI)		100 *ml* (30 gI)								

Iopromide (Ultravist) (370 mgI/*ml*)	Adult doses suggestion	41-162 *ml* for bolus IV injection	41-162 *ml*										
		81-162 *ml* for rapid IV infusion											

	Maximum dose	162 *ml* (60 gI)	162 *ml* (60 gI)										

Iopamiro (Iopamidol) (300 mgI/*ml*)	Adult doses suggestion	100-200 *ml*	100-200 *ml*	2.0-2.5 *ml*/Kg	50 *ml*				5-40 *ml* for femoral or subclavian		8-12 *ml*		
									25-50 *ml* for aorta for a distal runoff				

	Maximum dose	200 *ml* (60 gI)	200 *ml* (60 gI)						250 *ml*		90 *ml*		

Iopamiro (Iopamidol) (370 mgI/*ml*)	Adult doses suggestion	81-162 *ml*			40 *ml*	2-10 *ml*	25-50 *ml*	50 *ml*		50 *ml*-larger vessels			
		81-162 *ml*								10 *ml*-renal arteries			

	Maximum dose	200 *ml* (60 gI)	200 *ml* (60 gI)				200 *ml*	225 *ml*		225 *ml*			

Omnipaque (Iohexol) (300 mgI/*ml*)	Adult doses suggestion	50-200 *ml*	75-150 *ml*		200-350 mgI/Kg			30-90 *ml*		50-80 *ml*-aorta, 30- 60 *ml*branches, 5 -15 *ml*- renal arteries.	6-12 *ml*-Common carotid artery; 8-10 *ml*-Internal carotid artery; 6-9 *ml*-External carotid artery; 6-10 *ml* Vertebral artery.		

	Maximum dose									291 *ml*			

Omnipaque (Iohexol) (350 mgI/*ml*)		60-100 *ml*	350 *ml*		200-350 mgI/Kg	5 *ml* (3-14 *ml*)	40 *ml* (30-60 *ml*)	20-70 *ml*		50-80 *ml*-aorta, 30- 60 *ml*branches,5 -15 *ml*- renal arteries.			

	Maximum dose					Total combined-250 *ml*				250 *ml*			

Ioversol (Optiray) (320 mgI/*ml*)		25-75 *ml* (bolus injection)	50-150 *ml*		50-75 *ml*	8 *ml* (2-10 *ml*) for the left coronary; 6 *ml* (1-10 *ml*) for for the right coronary artery.						9 *ml* (6-15 *ml*)	

	Maximum dose	150 *ml*				250 *ml*						250 *ml*	

Ioversol (Optiray) (350 mgI/*ml*)		25-75 *ml* (bolus injection)			50-75 *ml*								50-100 *ml*

	Maximum dose	150 *ml*											250 *ml*

Iobitridol (Xenetix) (300 mgI/*ml*)					50-100 *ml*		30-60 *ml* (3-5 *ml*/Kg)						

	Maximum dose												

Iobitridol (Xenetix) (350 mgI/*ml*)		Depend on the organs under investigation, the diagnostic problem and, in particular, the different scan and image-reconstruction times of the scanners in use	1-1.5 *ml*/Kg	155-330 *ml*			30-60 *ml* (3-5 *ml*/Kg)	10-80 *ml*	105-205 *ml*				

	Maximum dose	1-1.5 *ml*/Kg						250 *ml*					

Iodixanol (Visipaque) (320 mgI/*ml*)		75-150 *ml*	75-150 *ml*		1 *ml*/Kg	20 *ml*							

	Maximum dose	150 *ml* (80 gI)	150 *ml* (80 gI)		100 *ml* (80 gI)

**Table 5:** Suggested single injection of adult doses and maximum total dose for non-ionic contrast media by Intrathecal route injection [31].

Non-ionic contrast media		Myelogram - cervical myelogram (*via* lumbar injection)	Myelogram - total columnar myelography	Myelogram -thoracic	Myelogram -spinal cord
Iopamiro (Iopamidol) (300 mgI/*ml*)	Adult doses suggestion	10 *ml*	10 *ml*		
	Maximum total dose				
Iohexol (Omnipaque) (300 mgI/*ml*)	Adult doses suggestion	4-10 *ml*		6-10 *ml*	6-10 *ml*
	Maximum total dose	3060 mgI		3060 mgI	3060 mgI

## 2. The adverse drug reaction (ADR) of iodinated contrast media and management

**Fig. 3:**
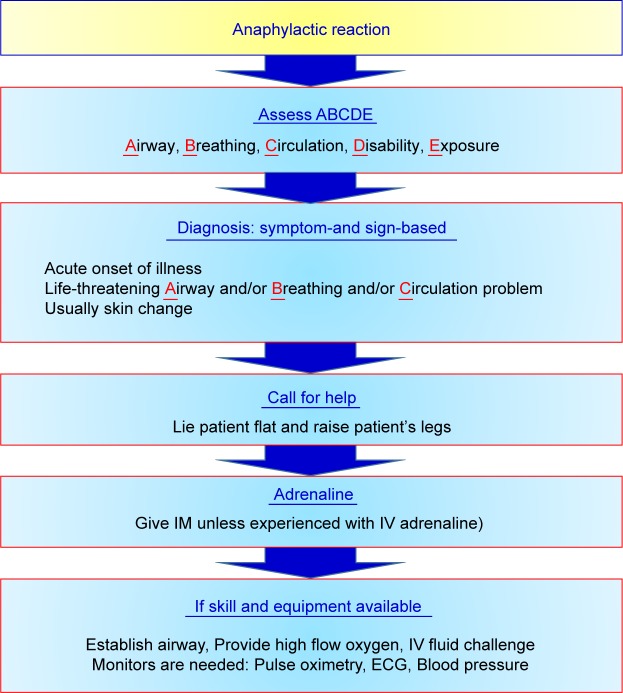
Advanced Cardiovascular Life Support (ACLS) guideline for the management and treatment of adverse effects on anaphylactic reaction.

**Fig. 4:**
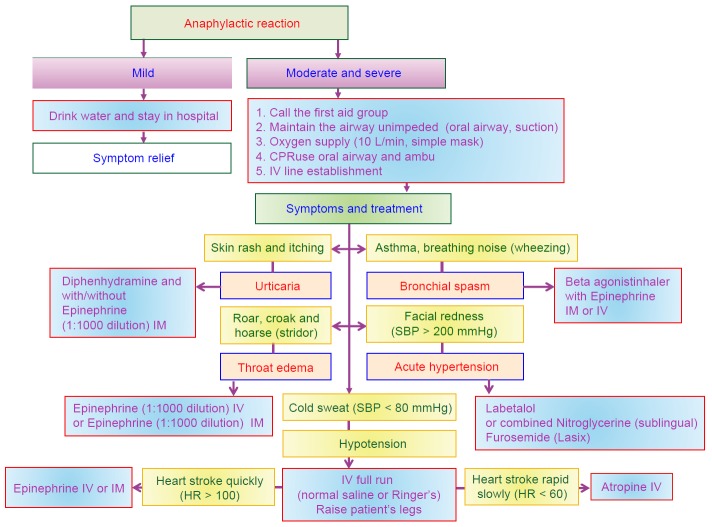
Management and treatment of anaphylactic reaction by iodinated CM is proposed in 2017 RSROC Contrast Media Manual.

ADR caused by iodinated CM includes chemical and constitutional effects. Chemical effects are mainly referred as contrast-induced nephropathy (CIN) and will be discussed later. Anaphylactic reaction is the most common situation in constitutional effect and may cause mild symptom such as nausea and vomiting, dizziness, rash and itch, or chest discomfort, shock in more severe situation, or even death [21, 23, 28, 29, 32]. Iodinated contrast media cause little allergic reactions, especially for low-osmolar contrast media (LOCM). The incidence of adverse effect to LOCM is 2 to 7/1000, that of severe allergic reaction to LOCM is lower 1 to 4/100,000, and that of lethal rate to LOCM is around 2-9/1000,000 [33, 34]. We should recognize adverse effects and receive early intervene to reverse bad situation. The management and treatment of adverse effects on anaphylactic reaction by Advanced Cardiovascular Life Support (ACLS) guideline is shown in Fig. 3. The Fig. 4 shows that management and treatment of anaphylactic reaction by iodinated CM is proposed in 2017 RSROC Contrast Media Manual [33]. There are several affecting factors for anaphylactic reaction by iodinated CM such as particularly allergy (arising from consuming sea foods or drugs), previous adverse reactions, history of asthma or bronchospasm, history of allergy, cardiac disease, dehydration, haematological and metabolic conditions (sickle cell anaemia, patients with thrombotic tendency), renal disease, neonates, old patients, anxiety and apprehension medications (β-blockers, interleukin-2 (IL-2), aspirin, NSAIDs) [33]. In addition, IOCM (ie, iodixanol (Visipaque^®^)) are associated with the highest risk of causing a delayed hypersensitivity reactions. The incidence of delayed hypersensitivity reactions to IOCM is 10.9% and 5%-6% for LOCM [33, 35, 36]. Lasser *et al.* suggested that two doses of corticosteroid prophylaxis (32 mg of methyl prednisolone, orally 12 and 24 h before iodinated CM injection signification reduced the iodinated CM-induce anaphylactic reaction [13, 34].

## 3. Molecular mechanism of contrast-induced nephropathy (CIN)

**Fig. 5:**
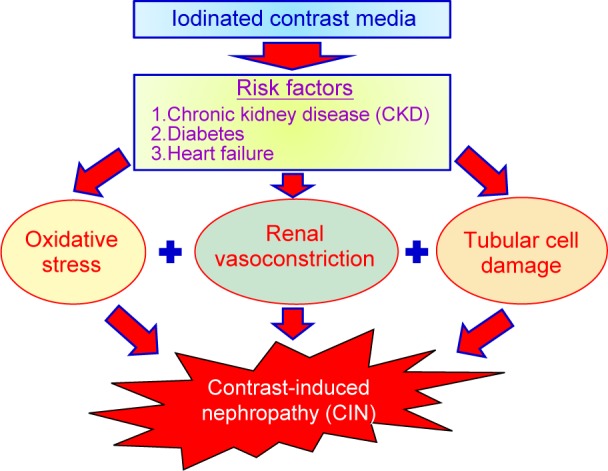
Three factors are responsible for contrast-induced nephropathy.

**Fig. 6:**
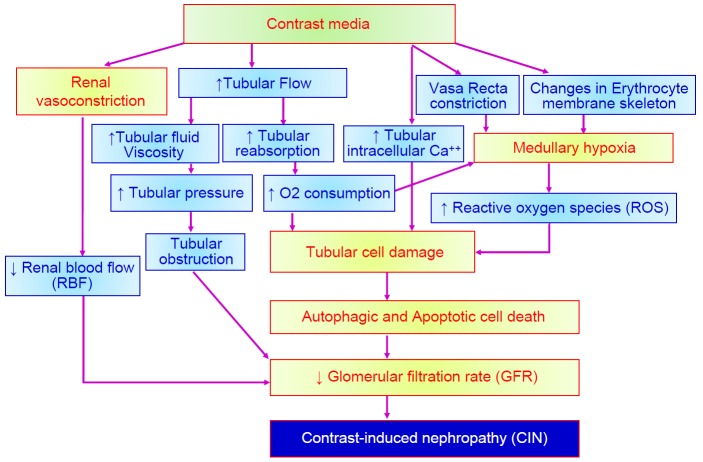
The detailed molecular mechanisms of contrast-induced nephropathy.

CIN is one of chemical adverse effects of iodinated CM. The pathophysiology of CIN is related to hemodynamic changes caused by vasoconstriction which makes a decrease of glomerular filtration rate (GFR) and a renal ischemia. Direct cytotoxicity to renal tubular cell is another pathway leading to kidney damage [37-49]. Norbert H. *et al.* medullary ischemia and direct cytotoxicity to renal tubular cell are two main mechanism to result in CIN. Medullary ischemia is a complex result of vasoconstriction, lower oxygen delivery and higher oxygen demand. In Fig. 5, there are three factors such as increasing oxidative stress, enhancing renal vasoconstriction and inducing tubular cell damage responsible for CIN [50, 51]. Several factors including renal ischemia, particularly in the medulla, reactive oxygen species (ROS) formation, reduction of nitric oxide production, tubular epithelial and vascular endothelial injury may be implicated in CIN. Many studies demonstrated that iodinated CM exert cytotoxic effects and renal tubular epithelial cells present severe cell death by autophagy and/or apoptosis [6]. Iodinated CM induces renal vasoconstriction by increase of adenosine and endothelin, and changes the blood flow from the medulla to the cortex and GFR are reduced. Reduction in renal blood flow can increase ROS release by oxidative stress. In tubular cells, iodinated CM directly caused osmotic necrosis or vacuolization leading to acute tubular cell death [15, 37-39]. Several antioxidant compounds have been demonstrated prevention effects by CIN, including sodium bicarbonate, N-acetylcysteine (NAC), ascorbic acid, statins, and recently, phosphodiesterase type 5 inhibitors [4-7]. The detailed molecular mechanisms of CIN are described in Fig. 6.

## 4. *In-vitro* studies on contrast-induced nephropathy (CIN) by iodinated CM.

In 2017 year, Charalampos Mamoulakis *et al.* summarize recent *in vivo* studies on oxidative stress related to CIN in animal models and humans [6]. Hereby, we summarize recent *in vitro* studies on the mechanisms in contrast-induced nephropathy (CIN). Direct damage, a risk factor of CIN, induces cell death to renal tubular cells caused by iodinated CM. Table 6 is a summary of the manifestation of CIN which is collected from *in vitro* studies. Inhibiting cell proliferation and inducing cell death are found in renal cell lines including KRK52-E, LLC-PK1, HKCS, HK-2 at the concentration higher than 75 mgI/*ml*. Importantly, iodinated CM induced cell death no matter whether in LOCM or IOCM. Apoptosis and/or autophagy are two cell types in cell death [52- 58]. Readers refer to our previous article for detailed molecular mechanisms of apoptosis and autophagy [59].

**Table 6:** *In vitro* studies of mechanisms on contrast-induced nephropathy (CIN) in iodinated contrast media.

In-vitro cell lines	Iodinated contrast media	Dose	Time of treatment	Results	References
KRK52-E (Rat kidney epithelial cell)	Iodixanol (Visipaque) Ioversol (Optiray) Iohexol (Omnipaque) Iopromide (Ultravist)	150 mgI/*ml*	0.5 h, 1h , 3 h , 6 h , 12 h , 24 h.	1. Decreasing cell proliferation by MTT assay. 2. Induce cells death by Trypan blue assay. 3. Increasing apoptosis by hematoxylin-stained.	[60]
NRK52-E (Rat tubular cells)	Iohexol (Omnipaque)	100 mgI/*ml*	24 h	1. Decreasing cell proliferation by MTT assay. 2. Increasing apoptotic cells by TUNEL assay. 3. Increasing caspase-3, caspase-9 and cytochrome c protein levels by western. 4. Decreasing cell viability by iohexol was aggravated with 3-MA pretreatment.	[61]
LLC-PK1 (Pig renal tubular epithelial cells)	Iohexol (Omnipaque) Iodixanol (Visipaque)	100 mgI/*ml*	24 h	1. Decreasing cell proliferation by MTT assay. 2. Increasing apoptotic cells by TUNEL assay. 3. Increasing caspase-8, caspase-9 and caspase-3 protein levels by western.	[62]
HK-2 (human embryonic proximal tubule)	Iopamiro (Iopamidol)	200 mgI/*ml*	0 h 12 h 24 h	1. Decreasing cell proliferation by MTT assay. 2. Increasing apoptotic cells by TUNEL assay. 3. The mRNA level of Bax was increased and Bcl-2 was decreased by qPCR. 4. Increasing Bax, caspase-3 protein levels and decreasing Bcl-2, HSP70 protein levels by western.	[63]
LLC-PK1 (Pig renal tubular epithelial cells)	Iodixanol (Visipaque)	4.7-75 mgI/*ml*	2h , 24h	1. Decreasing cell proliferation by MTT assay.	[58]
HK-2 (human embryonic proximal tubule)	Iopromide (Ultravist)	40 mgI/*ml* 20 mgI/*ml* 10 mgI/*ml*	24-72 h	1. Caused the breaking of intercellular connections and cell migration by scratch assay. 2. Increasing SGK, SNAIL1, CTGE, COL1A1 mRNA levels by qPCR	[64]
LLC-PK1 (Pig renal tubular epithelial cells)	Ioversol (Optiray)	100 mgI/*ml*	24 h	1. Increasing caspase-3 protein activity by caspase-3 activity assay	[56]
HK-2 (human embryonic proximal tubule)	Ioversol (Optiray)	100 μL/*ml* 200 μL/*ml*	24 h	1. Decreasing cell proliferation by MTT and LDH assay.	[55]
HK-2 (human embryonic proximal tubule)	Iodixanol (Visipaque)	25 mgI/*ml* 50 mgI/*ml* 100 mgI/*ml* 200 mgI/*ml*	2 h, 4 h, 8 h, 24h	1. Decreasing cell proliferation by CellTiter 96 assay.	[53]
LLC-PK1 (Pig renal tubular epithelial cells)	Iodixanol (Visipaque)	18.75-75 mgI/ *ml*	24 h	1. Decreasing cell proliferation by BrdU assay 2. Increasing apoptotic cells by cytoplasmic oligonucleosomes ELISA assay.	[52]

## 5. Conclusion

Autophagy and apoptosis were associated with the pathophysiology of CIN in *in vitro* reports. In conclusion, *in vitro* studies showed that increased cell death by apoptosis and/or autophagy was demonstrated in the kidney cell lines after the administration of iodinated CM. Inhibition of autophagy induced cell apoptosis suggested the protective role of autophagy in CIN. In the future, studies about how to reduce cellular stress and cell death by new methods or new compounds and understanding the details molecular mechanisms may be helpful for the development of new therapeutic strategies for the treatment of CIN.

## Declaration of Conflicting Interests

The author(s) declared no potential conflicts of interest with respect to the research, authorship, and/or publication of this article.

## Acknowledgments

This work was supported by the grant from China Medical University Hospital, Taichung, Taiwan (DMR-107-123). The authors also would like to express our gratitude to Miss Huei-Min Chen for drug information supports.
